# Supplementary material for: Autophagy Inhibits Intercellular Transport of Citrus Leaf Blotch Virus by Targeting Viral Movement Protein
Source: Viruses. 2021 Oct 30;13(11):2189. doi: 10.3390/v13112189 (PMC8619118; doi:10.3390/v13112189)
Supplement: Supplementary file 1 [file viruses-13-02189-s001.zip › viruses-1391493-supplementary.pdf]

viruses MDPI

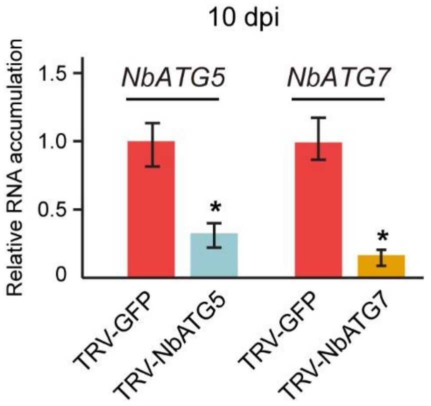

Figure S1. Relative mRNA accumulation of *NbATG5* and *NbATG7* following silencing using TRV–VIGS. Total RNAs were extracted from the upper leaves at 10 dpi. qRT–PCR was carried out using primer sets specific for *NbATG5*, *NbATG7* and *N. benthamiana* 18S rRNA as an internal control standard. The TRV–GFP sample was set to a value of 1.0. The asterisk indicates a significant difference ($P < 0.05$, Student's *t*test).

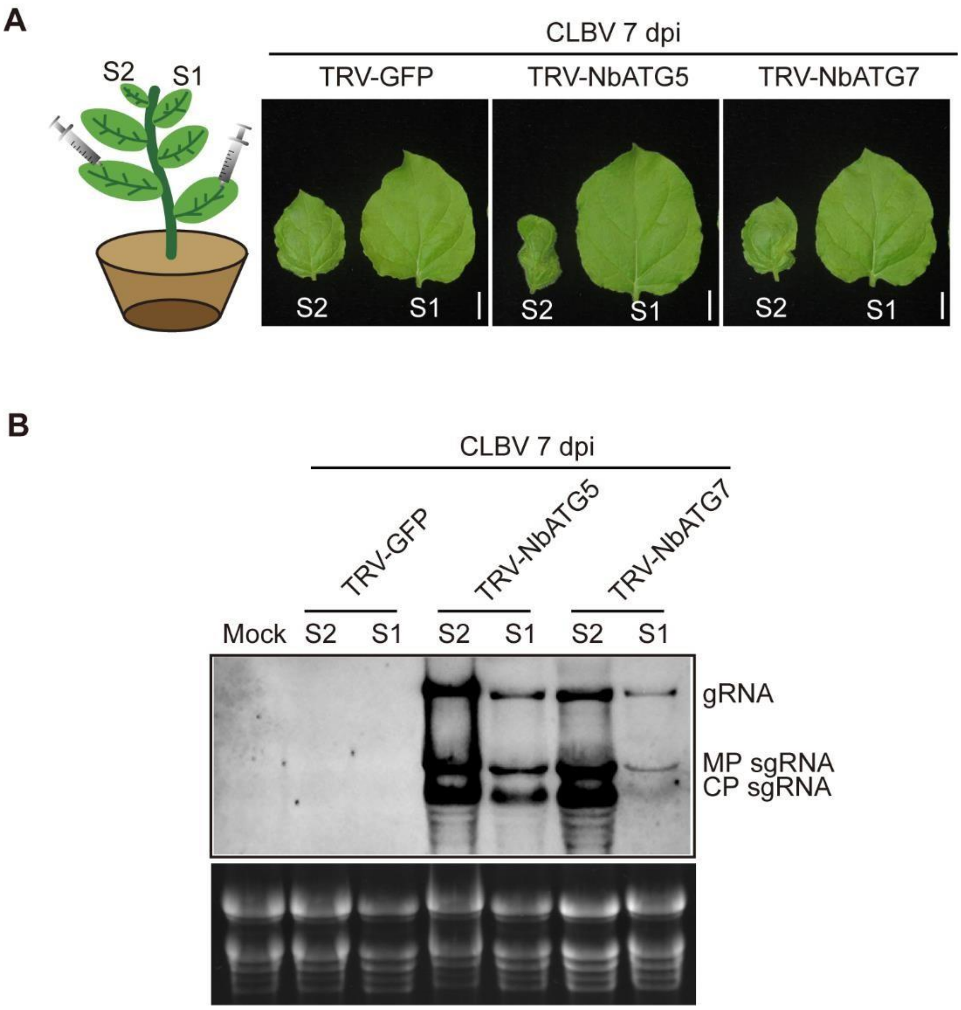

Figure S2. Effect of autophagy on CLBV systemic infection in *N. benthamiana*. **(A)** CLBV symptom expressions at 7 dpi on leaves of plants with *NbATG5* and *NbATG7* genes had been silenced using TRV–VIGS. Plants were inoculated with TRV–NbATG5 and TRV–NbATG7 or TRV–GFP as a control, and 10 days later, plants were inoculated with CLBV. The photographs show the upper two leaves as illustrated in the cartoon image in the left side of the panel. Scale bars, 1 cm. **(B)** CLBV RNA accumulation in infected plant at 7 dpi. Total RNAs were extracted from upper leaves

described in (A) and subjected to RNA blotting with a probe specific for CLBV genome. Ethidium bromide-stained 28S rRNA is shown as a loading control.

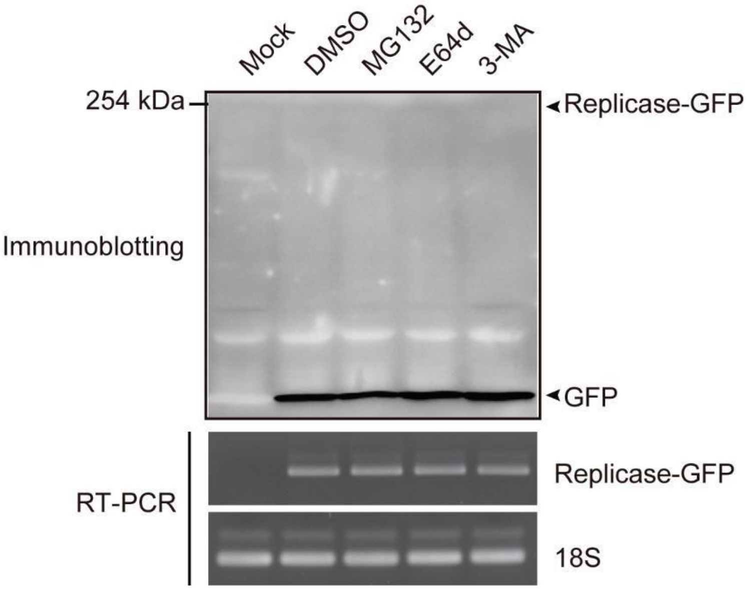

Figure S3. Protein and mRNA accumulations of transiently expressed. Replicase−GFP upon treatment with MG132, E64d and 3-MA.

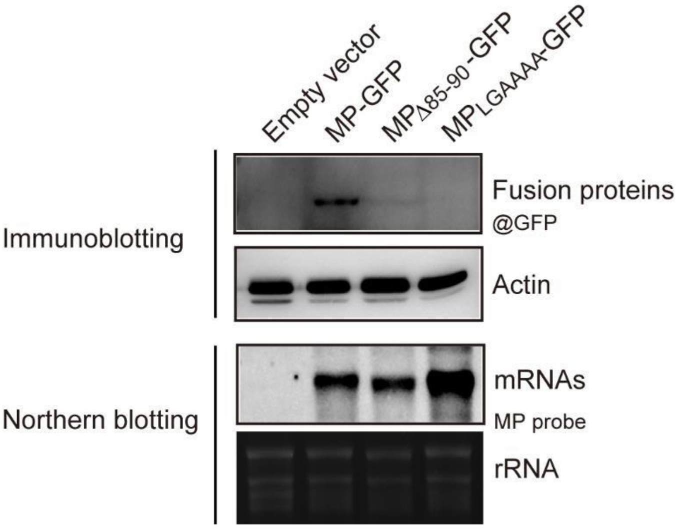

Figure S4. Protein and mRNA accumulations of transiently expressed CLBV MP with mutation in the AIM sequence fused with GFP.

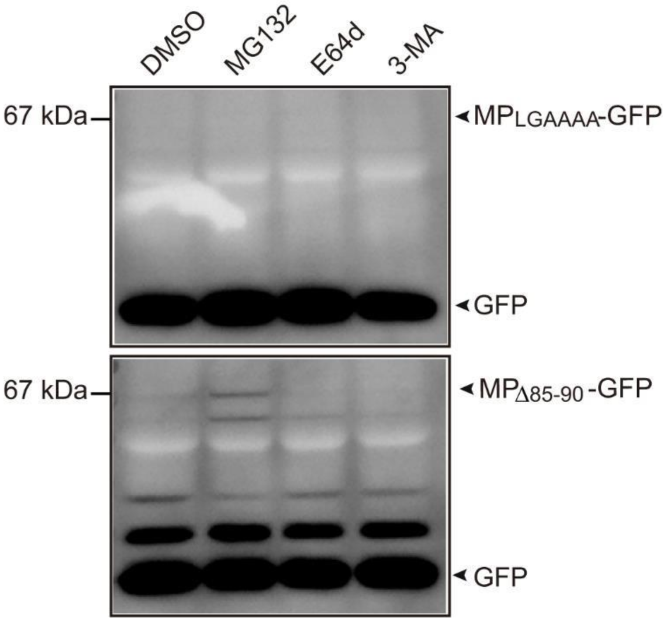

Figure S5. Protein accumulation of transiently expressed CLBV MP with mutation in the AIM sequence fused with GFP upon treatment with MG132, E64d and 3-MA.

Supplementary Table S1. A list of primers used in this study.

Primers used for construction of clbv infectious clone.

Clone Name	Primer Name	Oligonucleotide Sequence (5′–3′)
pCLBV	F-pCass-CLBV	agaggcctgggtaccGGAAAACGAAAGAAACCTACA
	R-CLBV	GGTCGGACCGCGAGGAGGTGGA- GATGCCATGCCGACCTTTTTTT TTTTTTTTTTTTTTTTTTTTTTC
	R-pCass-RZ	accgaattcgagctcCTTAGCCATCCGAGTGGAC- GTCCTCCTTCGGATGCC CAGGTCGGACCGCGAG- GAGGT
pCLBV-GFP	F-pCass-CLBV6526	cttgaattacatggagtcgacCTTAGGGAGGAGCGCTCTG- TAG
	R-pCass-CLBV8261	CTTAACATGGGACACATAATCCTTCTTACATCTC- TATGAGCTTAGC TTTATTTTT
	F-CLBV-GFP	GAAGGAT- TATGTGTCCCATGTTAAGTCTGAAACTGCGTCTTT GAA AAGAAGTCAGAGTGGAAGAGATTGGGG- CAATGATCGATTAAGAA AGTATGGTGAGCAAGGGCGAGGAGCTG
	R-CLBV-GFP	GGCCCGCCCTTCCACCCACGCCAGTTCACTT- GTACAGCTCGTCCA TGCCG
	F-pCass-CLBV-3T	ACTGGCGTGGGTGGAAGGGCGGGCC
	R-pCass-CLBV-3T	tgtcaaacactgatagtttaaacTGAAGGCGGGAAAC- GACAATCTGA
**Primers used for transient expression in *N. benthamiana***		
pCambia1302-Replicase -GFP	F_{speI}-Replicase	accatggtagatctgactagtATGGCTTT- GATGAGCAACAAAACTGC
	R_{speI}-Replicase	gctcaccatcctaggactagtAA- TATCTTCGTCTGAAGACTGTTC

pCambia1302-MP-GFP	F_{speI}-MP	accatggtagatctgactag-tATGGCTTCCCTCATCAATGTGAGC
	R_{speI}-MP	gctcaccatcctaggactagtCTT-GGTCCCAGTGTCGCTGGC
pCambia1302-MP-dAIM-GFP	F_{speI}-MP	accatggtagatctgactag-tATGGCTTCCCTCATCAATGTGAGC
	R-MPdAIM	GTGCAAGTAAGCAGCCTCTTTC
	F-MPdAIM	GAAAAGAGGCTGCTTACTT-GCACATAGCAATTAGATCATT-GCTGC CG
	R_{speI}-MP	gctcaccatcctaggactagtCTT-GGTCCCAGTGTCGCTGGC
pCambia1302-MP-*aim*-GFP	F_{speI}-MP	accatggtagatctgactag-tATGGCTTCCCTCATCAATGTGAGC
	R-MP-*aim*	ATCTAATTGC-TATTATTATTATTATAAGGTG-CAAGTAAGCAGCCTC TTTT
	F-MP-*aim*	CACCTTATAATAATAATAA-TAGCAATTAGATCATTGCTGCCG
	R_{speI}-MP	gctcaccatcctaggactagtCTT-GGTCCCAGTGTCGCTGGC
pCambia1302-CP-GFP	F_{speI}-CP	accatggtagatctgactag-tATGAAAATCAC-CAATGACAATGCC
	R_{speI}-CP	gctcaccatcctaggactagtCATCTC-TATGAGCTTAGCTTTATTTTT
pBin41-MP-HA	F_{BamHI}-MP	agcttcgactctagaggatccAC-CATGGCTTCCCTCATCAATGTGAGC
	R_{BamHI}-MP-HA	ttcgagctcgcccggggatccTCAAGCG-TAATCTGGAACATCGTATGGG-TACT TGGTCCCAGTGTCGCTGGC
pBin41-CP-HA	F_{BamHI}-CP	agcttcgactctagaggatccAC-CATGAAAATCAC-CAATGACAATGCC
	R_{BamHI}-CP-HA	ttcgagctcgcccggggatccTCAAGCG-TAATCTGGAACATCGTATGGG-TACA TCTC-TATGAGCTTAGCTTTATTTTT

pBI121-MP-mCherry	F_{BamHI}-MP	cacgggggactctagaggatccATGGCTTCCCTCATCAATGTGAGC
	R_{BamHI}-MP	tcaccatggtacccggggatccCTTGGTCCCAGTGTCGCTGGC
pBI121-CP-mCherry	F_{BamHI}-CP	cacgggggactctagaggatccATGAAAATCACCAATGACAATGCC
	R_{BamHI}-CP	tcaccatggtacccggggatccCATCTCTATGAGCTTAGCTTTATTTTT
pBin61-GFP-NbATG8c1	F_{BamHI}-NbATG8c1	acccccggggtcgacggatccATGGCGAAGAGTTCTTTCAAACTT
	R_{BamHI}-NbATG8c1	tctagttcatctagaggatccTTAATTGCCGAGCTCAAGAAACC
pBin61-GFP-NbATG8d	F_{BamHI}-NbATG8d	acccccggggtcgacggatccATGGCCGAAGCTGCTCGT
	R_{BamHI}-NbATG8d	tctagttcatctagaggatccTCAAGATTTGCAGAGAGAAAGCTG
pBin61-GFP-NbATG8f	F_{BamHI}-NbATG8f	acccccggggtcgacggatccATGGCAAAGAGTTCATTCAAGCA
	R_{BamHI}-NbATG8f	tctagttcatctagaggatccTCACACCAAGTTAAAGTCCCCAAATG
pBin61-GFP-NbATG8i	F_{BamHI}-NbATG8i	acccccggggtcgacggatccATGGGGAAGGCTTTCAAAAAA
	R_{BamHI}-NbATG8i	tctagttcatctagaggatccTCAACTATTTGCACGACCAAAGG
pBin61-eYFP-N-NbATG8c1	F_{BamHI}-NbATG8c1	cgccacaacatcgagggatccACCATGGCGAAGAGTTCTTTCAAACTT
	R_{SmaI}-NbATG8c1	gaattcgagctctatcccgggTTAATTGCCGAGCTCAAGAAACC
pBin61-eYFP-N-NbATG8d	F_{BamHI}-NbATG8d	acccccggggtcgacggatccATGGCCGAAGCTGCTCGT
	R_{SmaI}-NbATG8d	tctagttcatctagaggatccTCAAGATTTGCAGAGAGAAAGCTG
pBin61-eYFP-N-NbATG8f	F_{BamHI}-NbATG8f	acccccggggtcgacggatccATGGCAAAGAGTTCATTCAAGCA
	R_{SmaI}-NbATG8f	tctagttcatctagaggatccTCACACCAAGTTAAAGTCCCCAAATG
pBin61-eYFP-N-NbATG8i	F_{BamHI}-NbATG8i	acccccggggtcgacggatccATGGGGAAGGCTTTCAAAAAA
	R_{SmaI}-NbATG8i	tctagttcatctagaggatccTCAACTATTTGCACGACCAAAGG

pBin61-eYFP-C-MP	F_{BamHI}-MP/-N/-N84/-N90	gacgagctgtacaagggatccAC-CATGGCTTCCCTCATCAATGTGAGC
	R_{SmaI}-MP/-C	gaattcgagctctatcccgggTCACTT-GGTCCCAGTGTCGCTGGC
pBin61-eYFP-C-MP-N	R_{SmaI}-MP-N	gaattcgagctctatcccgggTCATAGAC-CTGTAATTATGGCAGCTACAGA
pBin61-eYFP-C-MP-C	F_{BamHI}-MP-C	gacgagctgtacaagggatccACCATGAC-CTGTACACCCACCAACAAAAT
pBin61-eYFP-C-MP-N84	R_{SmaI}-MP-N84	gaattcgagctctatcccgggTCAGTG-CAAGTAAGCAGCCTCTTTTCCG
pBin61-eYFP-C-MP-N90	R_{SmaI}-MP-N90	gaattcgagctc-tatcccgggTCATATTGGAACAAATCCAAGGTGCAAGTAA
**Primers used for bacterial expression**		
pMAL-MBP-MP	F_{XbaI}-MP/-N	tcagaattcggatcctcta-gaATGGCTTCCCTCATCAATGTGAGC
	$R_{HindIII}$-MP/-C	acgacggccagtgccaagcttTCACTT-GGTCCCAGTGTCGCTGGC
pMAL-MBP-MP-N	$R_{HindIII}$-MP-N	acgacggccagtgccaagcttTCATAGAC-CTGTAATTATGGCAGCTACAGA
pMAL-MBP-MP-C	F_{XbaI}-MP-C	tcagaattcggatcctctagaATGACCTG-TACACCCACCAACAAAAT
**Primers used for VIGs**		
pTRV-ATG5	F_{BamHI}-NbATG5	gtgagctcggtaccggatccATGG-GAAGTAAAGGGGCAGGAG
	R_{EcoRI}-NbATG5	tgagtaaggttaccgaattcAC-GTTCAGGTTCTGCACAAAGAAG
pTRV-ATG7	F_{BamHI}-NbATG7	gtgagctcggtaccggatccATGGCGGA-TAGTGGAAGAGGAAC
	R_{EcoRI}-NbATG7	tgagtaaggttaccgaattcCAACGTGTTT-GTATTGAGAAGA
**Primers used for RT-PCR**		
	F-CLBV CP-RT	ATGAAAATCAC-CAATGACAATGCC
	R-CLBV CP-RT	TTACATCTCTATGAGCTTAGC
	F-TRV CP-RT	GGTCCGATACGTCCTAATCCC
	R-TRV CP-RT	CCTAAGTAATTCGTGCATTGCG
**Primers used for RT-qPCR**		
	F-NbATG5-RT	GAA-GCTTATCTCCGAATCTCGTCTAAGC

	R-NbATG5-RT	CCAACTTTCAACTGCAGGTG-CATCTTG
	F-NbATG7-RT	AGGTCTCGATGTCTAATCCTC-TACGCCAG
	R-NbATG7-RT	AATCAAATCAGACAAATGTCTG-CAATCCTG
	F-CLBV-RT	AGCTGAGGCAGAGGATTTGA
	R-CLBV-RT	GAACTGCTGCAAATCGTTCA
	F-18SrRNA-RT	CGGCTACCACATCCAAGGAAGG
	R-18SrRNA-RT	GAGCTGGAATTACCGCGGCTG
**Primers used for Northern blot**		
	F-MP	ACCTGTACACCCACCAACAA
	R-MP	TTGCCCCAATCTCTTCCACT
	F-CWMV-3T	GCAGGGATTGACTCGTTGAT
	R-CWMV-3T	CGACTGCTTGACTAACACCC
